# Challenges in the management of a splenic pseudocyst by laparoscopic splenectomy in an adult patient: A case report

**DOI:** 10.1016/j.ijscr.2023.108718

**Published:** 2023-08-28

**Authors:** Natasha Rooksana Jeenah, Ramesh Damodaran Prabha, Harald Puhalla

**Affiliations:** Gold Coast University Hospital, 1 Hospital Blvd, Southport, QLD, Australia, 4215

**Keywords:** Splenic cysts, Splenic pseudocyst, Laparoscopic splenectomy, Cyst treatment, Cyst management

## Abstract

**Introduction and importance:**

Splenic cysts are classified as true cysts, or pseudocysts, and larger cysts tend to be symptomatic, requiring management which has evolved to include spleen-sparing procedures to minimize the risk of overwhelming post-splenectomy sepsis (OPSS) Pitiakoudis et al. (2011), Hansen and Moller (2004), Knook et al. (2019) [[1], [2], [3]]. Total splenectomy remains the gold standard management, and the importance of this case is the uncommon spontaneous occurrence of a pseudocyst, and the importance to pre-operatively consent and prepare the patient for total splenectomy would intra-operative conditions not allow for spleen-preserving techniques.

**Case presentation:**

CS, a 21-year-old lady, had two presentations to the emergency department with left upper quadrant abdominal pain. The only abnormality on assessment was a large splenic cyst on CT scan, which increased in size on re-presentation. She was consented for a splenic cyst fenestration, and for total splenectomy and optimized with vaccines would intra-operative conditions not allow for spleen-sparing. During the operation, the planes between the cyst and spleen parenchyma were ill-defined, and decision was made to proceed with total splenectomy to avoid bleeding complications. She recovered well, and was discharged 5 days post-operatively, and histology confirmed a pseudocyst (Fig. 1, Fig. 2).

**Clinical discussion:**

The management of splenic cysts remains difficult and with no clear guidelines to uniform treatment. There are multiple spleen-preserving techniques developed to avoid OPSS (Agha RA, Franchi T, Sohrabi C, Mathew G, for the SCARE Group, 2020 [4]), however management remains individualized and case-specific.

**Conclusion:**

Pseudocysts can occur without splenic trauma or infarct. Management is case-based, and patients with large symptomatic cysts should be consented and prepared for total splenectomy would conditions not be safe for spleen-preserving interventions.

## Highlights

• Splenic pseudocysts can occur without trauma and are diagnosed on histology
• Size and location of splenic cysts, along with patient's symptoms guide decision for intervention
• Spleen-preserving interventions avoid overwhelming post-splenectomy sepsis
• Spleen-preserving techniques should be considered in carefully chosen patients
• Total splenectomy is the golden standard for splenic cysts management and patients should be consented and prepared for it pre-operatively would spleen-sparing procedures be abandoned intra-operatively

## 1 Introduction

Splenic cysts are an uncommon surgical encounter and are classified as either true (primary) cysts or pseudocysts (secondary) through various systems, including the Fowler's, Martin's, Morgenstern's, and Altemeier classifications. True cysts have an epithelial lining and can be further sub-classified as parasitic or non-parasitic cysts, whereas the latter can be grouped as congenital or neoplastic cysts [1,2]. As for pseudocysts, they usually result from a splenic trauma or splenic infarct, including degeneration secondary to radiological intervention for the management of a splenic bleed.

**Fig. 1:**
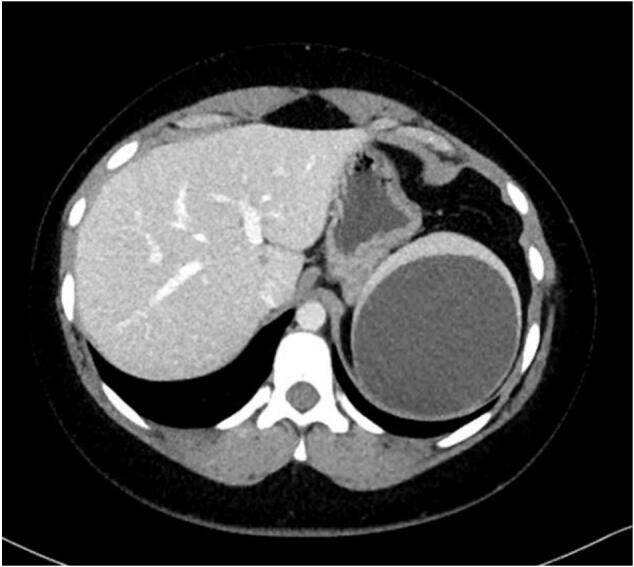
Axial view of the splenic cyst on CS CT scan.

**Fig. 2:**
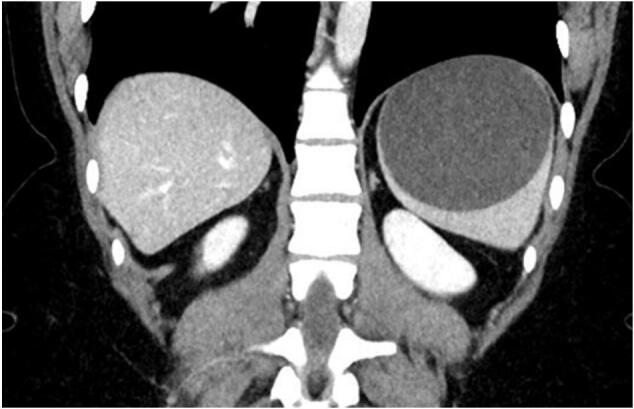
Sagittal view of the splenic cyst on CS CT scan.

The increasing use of abdominal ultrasound and CT scans has resulted in an increase in the incidence of splenic cysts [3]. Most of those incidental cysts identified on abdominal imaging require no intervention, while larger ones may present with left upper quadrant pain and require intervention. Even though literature is rich regarding splenic cysts classification and pathology, their management remains individualized and case-specific. Total splenectomy is the gold standard management for those larger, symptomatic, and complicated cysts, and more recently, spleen-sparing approaches have been employed to minimize the risk of overwhelming post-splenectomy sepsis. Also, this paper has adequately followed all the guidelines provided by the SCARE checklist [4]**.**

## 2 Presentation of case

A twenty-one-year-old lady, CS, was referred to the surgical outpatient clinics with a large symptomatic splenic cyst. She was a university business student and had previously presented to the emergency department with four months of recurrent, self-limiting left upper quadrant pain. She experienced no other gastrointestinal symptoms or other constitutional symptoms, and she had no other significant past medical history or trauma. Her vital signs were within normal range, her abdomen was soft and non-tender, with no palpable mass. The splenic cyst was initially diagnosed on abdominal ultrasound and was measured as 8 × 8.5 × 8.8 cm. A CT scan organized 2 weeks later, due to an increase in the severity of the pain, confirmed an intact splenic cyst, but larger in size (measuring 8.9 × 8.7 × 8.1 cm). It contained simple fluid and no other intra-abdominal pathology.

CS was booked and consented for a laparoscopic splenic cyst fenestration, and for a total splenectomy would the intra-operative conditions not allow for successful spleen preservation. Vaccine prophylaxis was performed 2 weeks pre-operatively as per current guidelines of Spleen Australia; that is an organisation that aims to guide people to prevent infectious diseases via comprehensive guidelines and recommendations [5]. For the procedure, CS was supine, the splenic flexure was mobilized, congenital omental and colonic adhesions were released, and the short gastric vessels secured with the LigaSure vessel sealing system. On assessment, the planes between the cyst and the spleen parenchyma were difficult to define, and the intra-operative decision was to proceed with laparoscopic total splenectomy which was uneventful. A 19Fr Blake drain was left in the splenic bed, and the patient was returned to the ward for observation. CS had an uncomplicated recovery, the drain and serum lipase were normal. The drain was removed on day 4, and she was discharged on day 5 after being registered with Spleen Australia for ongoing vaccinations. Histology confirmed a pseudocyst of the spleen and normal splenic parenchyma with no evidence of malignancy. She had made full recovery when reviewed at the outpatient appointment in 4 weeks.

## 3 Clinical discussion.

Spleen-preserving interventions include partial splenectomy, total cystectomy, marsupialization or cyst decapsulation or unroofing, and have been developed to avoid OPSS [6]. Marsupialization or percutaneous drainage of a cyst tend to result in higher risk of infection or disease recurrence through re-accumulation of fluid, bleeding, and increased risk of inflammation and adhesion [7,8]. As for the use of sclerosant injection into the cyst, pain and fever are reported, as well as recurrence requiring repeated therapy to achieve complete resolution [7,9]. Partial splenectomy, leaving as little as 25 %, has proved sufficient for adequate immunological function; however, it also carries a risk of intra-operative bleeding [8].

Spleen preserving technique was considered for CS; however, an intra-operative decision for total splenectomy was made for several reasons. Firstly, marsupialization or fenestration, as discussed pre-operatively with CS, was abandoned as the cyst was too deeply embedded into the spleen and would have increased the risk of bleeding if attempted. A recently reported successful case of splenic cyst fenestration involved a superficial cyst located at the pole [10]. In our case, cystectomy was also not possible as the planes between the cyst and the spleen were poorly demarcated, and because of the large size of the cyst. Partial splenectomy was not attempted as the section of the spleen involved was too close to the splenic artery, carrying a high risk of hemorrhage.

The management of splenic cysts remains difficult, and there are no clear guidelines for uniform management. The article by Karfis et al. [11] provides a review of the surgical management of non-parasitic splenic cysts in adults. The authors discuss the various surgical techniques available, including laparoscopic fenestration and total splenectomy. The article highlights the challenges in the management of splenic cysts, including the need to accurately diagnose the type of cyst and the potential for complications during surgery. The authors conclude by emphasizing the importance of individualized treatment planning and close collaboration between the surgeon and other medical specialists in managing splenic cysts in adults. Overall, the literature recommends monitoring for cysts less than 5 cm in size, and spleen-preserving techniques are advocated with the aim of preserving immunological and hematopoietic functions. However, one major risk is cyst recurrence or new cysts formation post surgery. In some cases, repeat surgeries may be required to address these new cysts. Spleen-preserving techniques are also high-risk and may cause damage to the spleen or impair its normal functions.

On balance, it appears to be reasonable to aim for a spleen-sparing technique, depending on the cyst size and location, but also on the availability of expertise and technology as well as consent and preparation for a total splenectomy would the latter be a safer option. Despite increasing incidence, the literature lacks large studies on the management of non-parasitic splenic cysts in adults comparing short and long-term outcomes of treatment options. Also, the current literature is limited in its ability to provide clear guidance for treatment teams regarding the best approach for long-term patient outcomes. This is due, in part, to a lack of large, well-designed studies that compare the short and long-term outcomes of different treatment options. Currently, the available literature provides conflicting results and limited data on the long-term outcomes of different treatment options. As a result, there is a need for further research to better understand the best approach to the management of non-parasitic splenic cysts, for example, large, controlled studies that compare the outcomes of different spleen-preserving techniques and total splenectomy. This research should consider factors such as patient outcomes, quality of life, and long-term safety. Additionally, there is a need for studies that compare the short- and long-term outcomes of different approaches, such as the use of imaging or other diagnostic tests to monitor the growth of small cysts. Further studies are warranted to guide treating teams for the best possible long-term patient outcome. For now, as explicated in this case, patients with large symptomatic splenic cysts should be consented both for spleen-sparing surgery, and for total splenectomy would this be a safer option.

## 4 Conclusion

Splenic pseudocyst can occur without trauma, and are diagnosed by histology. There are severel spleen-sparing procedures that can be employed to minimize the risk of OPSS depending on the size and location of the cyst. Further studies are required for more uniform management, though patients should always be consented for total splenectomy would circumstances not allow for spleen sparing procedure.

## Author contribution

1. Dr Natasha Rooksana Jeenah - Author, manuscript write up, data analysis and interpretation

2. Dr Ramesh Damodaran Prabha - Study concept

3. Dr Harald Puhalla - Second reader

## Informed consent

Written informed consent was obtained from the patient for publication of this case report and accompanying images. A copy of the written consent is available for review by the Editor-in-Chief of this journal on request.

## Sources of funding

None.

## Ethical approval

Ethics approval is exempt at our institution.

## Provenance and peer review

Not commissioned, externally peer-reviewed.

## Declaration of competing interest

None to declare.
